# Graphite, graphene on SiC, and graphene nanoribbons: Calculated images with a numerical FM-AFM

**DOI:** 10.3762/bjnano.3.34

**Published:** 2012-04-02

**Authors:** Fabien Castanié, Laurent Nony, Sébastien Gauthier, Xavier Bouju

**Affiliations:** 1 CEMES-CNRS, Centre d’élaboration des matériaux et d’études structurales, 29 rue Jeanne-Marvig, BP 94347, F-31055 Toulouse Cedex 4, France; 2 Université de Toulouse, UPS, 29 rue Jeanne-Marvig, BP 94347, F-31055 Toulouse Cedex 4, France; 3 Aix Marseille Université, IM2NP, Centre scientifique de Saint-Jérôme, Service 151, Avenue Escadrille Normandie-Niemen, F-13397 Marseille Cedex 20, France; 4 CNRS, IM2NP (UMR 7334), Marseille, France

**Keywords:** calculations, FM-AFM, graphene, graphite, image, nanoribbon

## Abstract

**Background:** Characterization at the atomic scale is becoming an achievable task for FM-AFM users equipped, for example, with a qPlus sensor. Nevertheless, calculations are necessary to fully interpret experimental images in some specific cases. In this context, we developed a numerical AFM (n-AFM) able to be used in different modes and under different usage conditions.

**Results:** Here, we tackled FM-AFM image calculations of three types of graphitic structures, namely a graphite surface, a graphene sheet on a silicon carbide substrate with a Si-terminated surface, and finally, a graphene nanoribbon. We compared static structures, meaning that all the tip and sample atoms are kept frozen in their equilibrium position, with dynamic systems, obtained with a molecular dynamics module allowing all the atoms to move freely during the probe oscillations.

**Conclusion:** We found a very good agreement with experimental graphite and graphene images. The imaging process for the deposited nanoribbon demonstrates the stability of our n-AFM to image a non-perfectly planar substrate exhibiting a geometrical step as well as a material step.

## Introduction

In the family of atomic force microscopy (AFM) techniques, the frequency-modulation (FM) mode provides subatomic and submolecular resolution [1–4]. Since the pionnering work performed by Giessibl [5], a large variety of surfaces have been observed at the atomic scale. For example, atomic features were imaged on Si [6–12], InSb [13], GaAs [14], Ge [15], NiAl [16–17], MgO [18–20], NaCl [21–25], CaCO_3_ [26], TiO_2_ [27–29], NiO [30], KBr [21,31–35], CaF_2_ [36], and graphite [37–44] to mention just a few. Moreover, from monolayer to single molecules, submolecular resolution has been obtained on various molecular systems [3,45–59]. Recently, impressive results were shown with single pentacene C_22_H_14_ and C_16_H_10_N_2_O_2_ molecules adsorbed on a thin NaCl film deposited on a Cu(111) surface [52–53]. These breakthroughs were possible with a functionalized tip, that is, with a CO molecule attached to the tip apex acting as a supertip [60]. Most of the mentioned studies were based on a technical improvement consisting of the use of a tuning fork of the qPlus sensor type [61]. This sensor is an AFM tip that is fixed to one branch of a quartz tuning fork and provides a stiff probe capable of being approached close enough to the sample without touching the surface [62]. When the probe is oscillating above the sample, one of the characteristics of an experimental FM-AFM setup is the presence of several feedback loops to pilot the probe based on the dynamic behavior of the oscillator. Briefly speaking, an important element of the FM-AFM experimental apparatus is the frequency detection by demodulation performed with the aid of a phase-locked loop (PLL). This allows measurement of the frequency shift Δ*f* from the fundamental resonance frequency of the free cantilever due to the tip–sample interactions. Moreover, two controllers are involved in the FM-AFM, namely the amplitude-controller (AC) and the distance-controller (DC) modules. The first one deals with the control of the oscillation amplitude of the probe, maintaining it at a constant value, and giving at the end an image representing a dissipation measurement. The second one keeps the resonance frequency shift Δ*f* due to the probe–surface interaction constant, hence providing the topographic image. The complexity of the two entangled loops of the FM-AFM, each with different gain parameters to be adapted to the experimental conditions, has been tackled through analytical and numerical solutions. Different approaches have been proposed to theoretically describe the FM-AFM [63–66]. The numerical FM-AFM described in this paper is based on the development already described by Nony et al. [67], and the details of the adaptation and the improvement will be described elsewhere [68]. All the blocks constituting the experimental FM-AFM setup were translated into numerical blocks in the overall n-AFM.

Graphene is a material that is now widely tackled in the condensed-matter community due to its fascinating prospects related to its particular electronic properties [69–72]. Many papers report on the growth process, which occurs mainly on metallic surfaces or on the silicon carbide surface, and on the characterization at the atomic scale by scanning tunneling microscopy (STM) or Raman spectroscopy [73–79]. Recently, the ability to create nanoribbons of graphene [80–82] arises because such a system exhibits a gap opening, thus providing a semiconducting behavior to the material. Actually, the structure of the edge of these nanoribbons of few nanometers in width plays a role in the expected electronic properties due to the confinement effect and due to the reactivity of the carbon atoms at the edges [79,83]. It is thus important to control and to determine the atomic structure of these edges, especially if one wants to functionalize them with molecules to tune their electronic properties [83].

Here, we propose a reliable numerical FM-AFM tool to study the imaging process with a good flexibility in terms of parameter choice. The efficiency of this numerical AFM is showed through model systems of three graphitic structures, namely a graphite substrate, a graphene surface on a SiC substrate, and the edges of graphene nanoribbons, in frozen-atom and free-atom modes.

## Technical details of the numerical AFM (n-AFM)

### n-AFM in frequency-modulation mode

The n-AFM simulates the behavior of a frequency-modulation AFM with parameters compatible with an ultrahigh-vacuum environment. The probe oscillates at or close to its fundamental resonance frequency 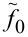. In this mode, the amplitude of oscillation is kept constant. When the oscillator is far enough from the sample, it can be considered as a free oscillator with *f*_0_ = 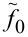. Upon approach toward the sample, an interaction between the tip and the sample appears and disturbs the oscillator motion, which leads to an almost instantaneous frequency shift, Δ*f* = 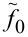 − *f*_0_. The frequency shift varies depending on the tip–sample distance. This is a critical parameter of FM-AFM.

As already mentioned and described in previous contributions [65,67,84], a FM-AFM is composed of several blocks dedicated to AC and DC modules, and also the PLL. The PLL block has as input signal, i.e., the normalized signal depicting the oscillator motion. This block is used as a frequency demodulator and as a synchronized signal generator. Indeed, the input signal is demodulated in order to compute the frequency shift and a signal synchronized with the input is generated in order to be used as the new normalized excitation signal. In this way, the excitation signal remains coherent with the oscillation of the probe.

The AC keeps the oscillation amplitude constant and equal to a predefined setpoint. Large-amplitude (typically 10–20 nm) to small-amplitude (of the order of 0.02 nm to mimic a qPlus sensor [62]) settings are available with the n-AFM. The DC allows the regulation of the tip–sample distance based on minimizing the difference between Δ*f* and the frequency setpoint. This regulation yields the sample topography. Each block was transposed into a numerical program and included in a general code written in Fortran 90 language. Just a few parameters are needed as input for the oscillator: stiffness constant *k*, quality factor Q, resonance frequency *f*_0_, amplitude *A*.

**Figure 1:**
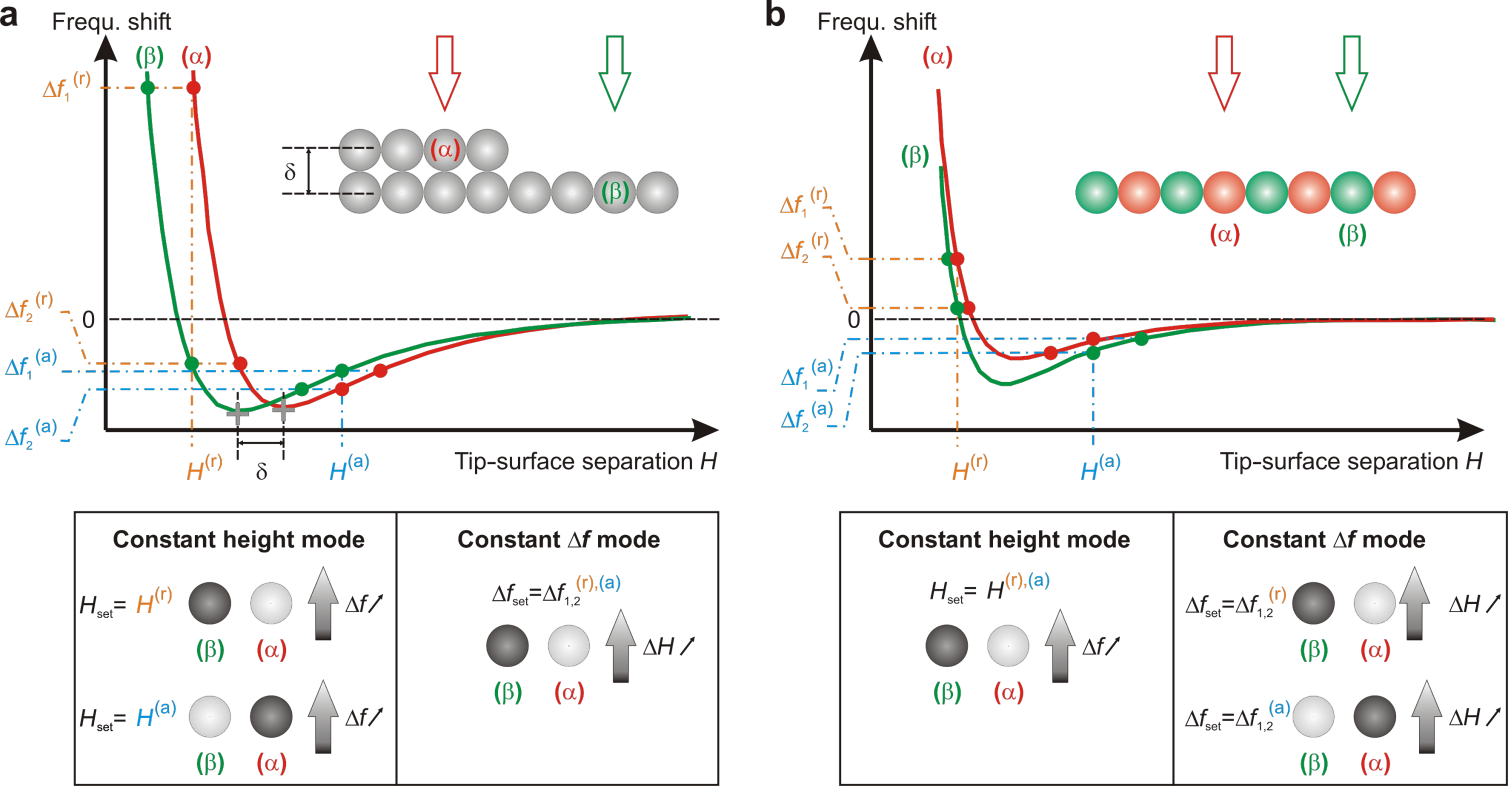
Illustration of constant-height and constant-Δ*f* imaging modes in nc-AFM. We consider, as an illustrative situation, the cases of a homogeneous and atomically corrugated surface with a step (a) and a heterogeneous, but still atomically corrugated surface (b). In each situation frequency shift versus tip–surface separation (*H*) curves are presented above α and β surface sites. The tables below the curves illustrate the resulting imaging contrast (from white to dark grey, standing for large to small values of the variable) above each type of site depending upon the imaging mode. Contrast-inversion situations may appear depending on (i) the imaging mode, (ii) the interaction regime between the tip and the surface and (iii) the nature of the interaction between the tip and the surface atoms.

The versatility of the n-AFM allows the production of two types of image. In constant-height mode, the tip is approached toward the surface up to the point where the predefined setpoint, Δ*f*_set_, corresponding to a tip–surface separation, *H*_set_, is reached. Then, the DC is disengaged, the *XY*-scan is engaged and the Δ*f* variations around Δ*f*_set_ are recorded. In this situation, the scan is therefore performed at nearly constant height, *H*_set_ (subject to vertical drift, which is obviously to be reduced as much as possible). Conversely, in constant-Δ*f* mode, the DC remains engaged. Then, the image depicts the regulation of the tip–surface separation that is required to maintain constant Δ*f*_set_. Therefore, beyond the known influence of (i) the tip–surface interaction regime (attractive versus repulsive; the attractive regime is such that *H* > *H*_min_, and the repulsive regime is such that *H* < *H*_min_, where *H*_min_ corresponds to *H* at Δ*f*_min_, i.e., the minimum in the frequency shift versus tip–surface separation (*H*) curve) and (ii) the chemical nature of the tip–surface interaction on the contrast formation of the resulting images, the measured images may as well depend on the acquisition mode. To illustrate this point, let us consider two situations illustrated in Figure 1.

First, we consider a reactive or inert tip interacting with two identical atoms, one on an atomically flat lower terrace, and the other on a nearby upper terrace. Owing to the similar chemical nature of the atoms, Δ*f* versus *H* curves measured on top of each of them exhibit similar features, and notably similar Δ*f* minima. The curves are simply *H*-shifted with respect to each other (Figure 1a). This will correspond to the case of the reconstructed graphene discussed hereafter. Second, we consider a less reactive or inert tip above two different surface sites lying at almost the same height on the surface (e.g., a top and hollow site on a graphite surface, as explained hereafter, Figure 1b). In the case of the corrugated surface, it can be seen in Figure 1a that an image recorded in constant-height mode in the attractive regime will yield an inverted contrast compared to an image recorded in constant-Δ*f* mode, whatever the value of Δ*f*_set_ is (attractive or repulsive regime). In the case of the heterogeneous surface, an inversion contrast will be observed between a constant-Δ*f* mode image acquired in the attractive regime and a constant-height mode image, whatever the interaction regime is.

Moreover, a molecular dynamics (MD) module is added by linking the n-AFM to the MD code DL_POLY [85]. This MD module can be implemented when it is necessary to take temperature conditions and/or deformations of the tip and the sample upon interaction into account. One of the main difficulties here is to handle the different time scales that characterize the different dynamic behaviors of the oscillator and the AFM junction atoms. Finally, a Kelvin probe force microscopy module (KPFM) [86–88] will be included in a near future. It should be mentioned also that when the tip interacts chemically with the substrate through bond creation between the tip apex atom and surface atoms, the choice of the force-field method may be difficult to justify. In that case, although reactive force fields exist [89–91] and may be implemented with the n-AFM, advanced first-principles methods [92] are well adapted to deal with local changes of electronic structure when the tip interacts with the sample surface, especially for KPFM [93–94]. For weak chemical interactions and van der Waals forces, theoretical studies have demonstrated accurate results for carbon-based systems [95–97], but are too slow and too computationally expensive compared to semiempirical models in the context of the n-AFM. The overall n-AFM system will be described elsewhere [68].

The settings used for this study are similar to the ones in [52], which correspond to a qPlus sensor: *A*_set_ = 0.2 Å, *f*_0_ = 23165 Hz, *k_c_* = 1800 N·m^−1^, Q = 50000.

An important input is the tip–sample interaction, which will be described in the following section for the three graphitic structures.

### Description of the interaction forces

In this study, the used model for the tip is composed of a nanosphere to mimic the probe body supporting a cluster of atoms for the tip apex. The sphere has a radius *R* of 4 nm and its force of interaction with a surface 
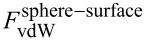
 is well described by

[1]
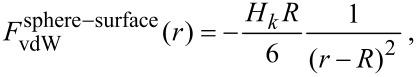


if (*r* − *R*) « *R* [98]. *H_k_* is the Hamaker constant (1 eV) and *r* the sphere–surface distance.

The cluster has a pyramidal diamond-like structure and is composed of 29 atoms [99]. The external interactions, that is between the atoms of the tip cluster and the atoms of the sample, are described by a Buckingham pairwise potential:

[2]
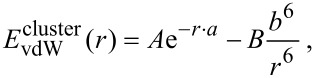


where *a*, *b*, *A* and *B* are constants depending on the type of atoms and are chosen in the data file of the MM4 force field [100–101].

When the MD module is switched on, the atoms of the tip and of the substrate are free to move under the constraints of internal and external interactions, and of a thermostat accounting for the external temperature. This constitutes the so-called free-atoms mode. In this case, the *n*-body Tersoff potential [89,102] was used for the internal interactions between atoms of each of the subsystems (the tip apex and the sample). This potential is designed to reproduce the covalent systems of the group IV elements in the periodic table (carbon, silicon, germanium, etc). Recent improvements of this potential [103] do not modify the results presented below.

In the case of graphite, the van der Waals interaction between two layers is described by a standard Lennard-Jones potential:

[3]
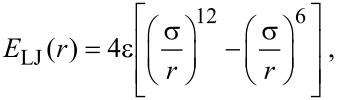


with ε = 0.011 eV and σ = 3.2963 Å.

## Results and Discussion

### Graphite surface imaging

AFM imaging of the graphite surface is a difficult task because the interaction between the tip and the sample is generally weak. Following Hembacher et al. [41], the normalized frequency shift γ = *kA*^3/2^Δ*f*/*f*_0_ is estimated to be |γ| < 1 fN·m^1/2^. With the parameters used with the n-AFM, one gets γ ≈ 7 × 10^−18^ Δ*f* N·m^1/2^, that is γ ≈ −0.1 fN·m^1/2^ with Δ*f* = −13 Hz. From an experimental point of view, such a low value explains why graphite imaging is tedious and so challenging.

There are several previous studies that tackle the atomic determination of the graphite surface by FM-AFM [37–44,104–106]. Indeed, there is a discrepancy in the interpretation of the brightest features on the surface. By coupling STM and FM-AFM [41,44], one may identify the actual graphite structure observed in the images. Nevertheless, the role played by the tip (structure and composition) seems to impact directly upon the imaging process [44,107–108].

**Figure 2:**
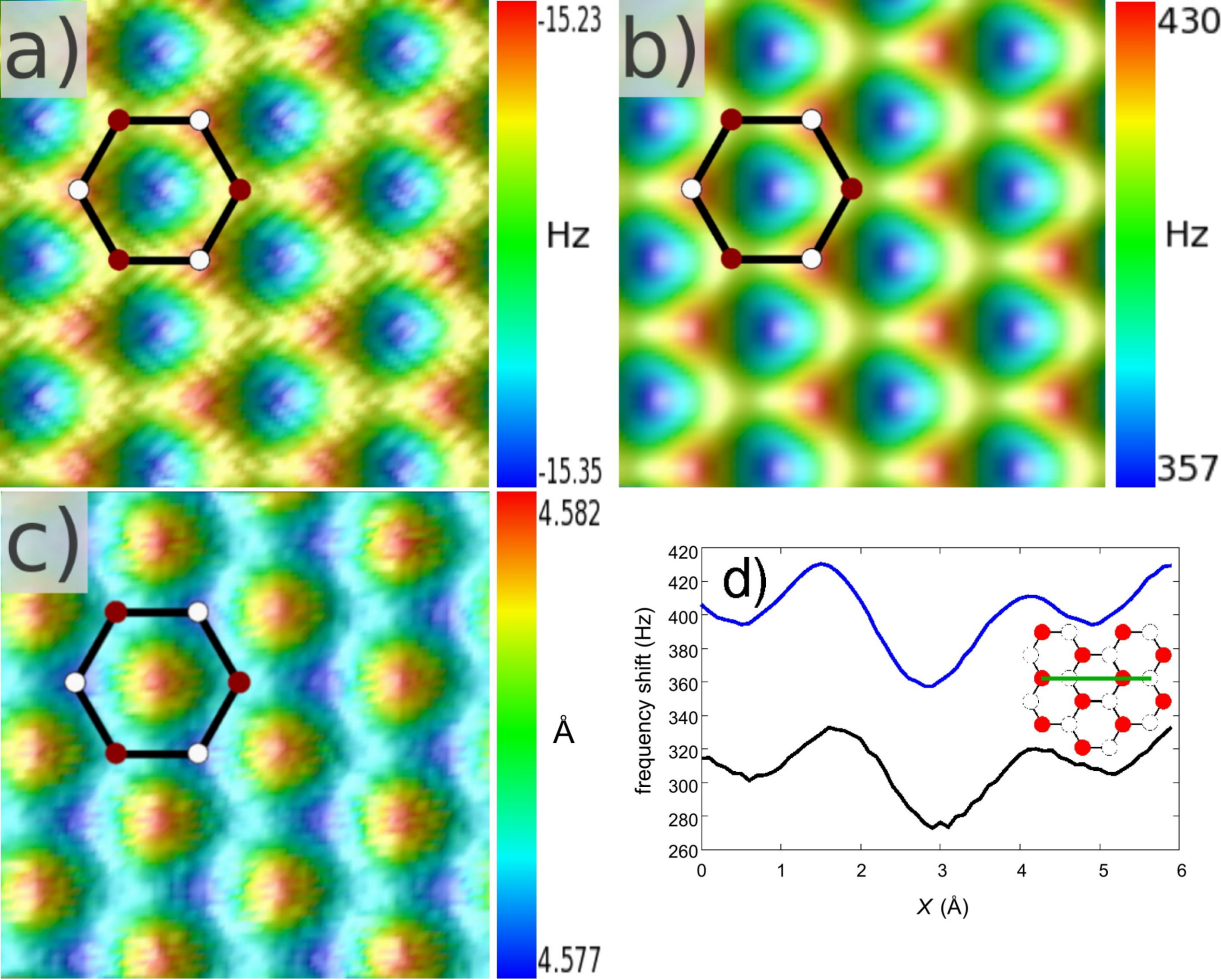
(a) Constant-height FM-AFM image of the graphite surface with *H*_set_ = 4.3 Å. White and dark circles correspond to *A* and *B* atoms, respectively. (b) Same as in (a) with *H*_set_ = 2.75 Å. (c) Constant-frequency-shift FM-AFM image of the graphite surface with Δ*f*_set_ = −13 Hz. The size of these three images is 9 × 9 Å^2^. (d) Scanlines above the green line shown in inset in the constant-height mode (*H*_set_ = 2.75 Å) for the frozen-atoms (blue) and free-atoms (black) regimes.

Here, we consider a graphite (0001) sample consisting of three graphene layers stacked with the abab structure each separated by 3.34 Å [109] and with 1792 carbon atoms each. The carbon atoms on the uppermost layer of the sample may be classified into two types, *A* with another neighboring atom just underneath, and *B* above a hollow site *h*. The tip is composed of 29 carbon atoms with a diamond-like organization. Results are presented in Figure 2 (in all the presented images, the scanning is from the left to right alternately, and from the bottom to the top). Force–distance spectra above different surface sites show a rather small variation due to the softness of the interaction, as sketched and enhanced in Figure 1b and similar to the Figure 2c (black and open symbols) in [44]. Figure 2a shows an image of the graphite surface in the frozen-atoms mode and at constant height, *H*_set_ = 4.3 Å, where *H* is the distance between the topmost surface plane and the terminating atom of the tip apex. At this distance, the tip oscillates in the attractive part of the tip–surface interaction force curve. This is the reason why the frequency shift exhibits a negative value. The maximum of corrugation is about 0.12 Hz, which is very weak. Such a low value is measured with the n-AFM because it works ideally without external noise sources and with no atomic vibration in the frozen-atoms mode. The *A* atoms appear brighter than the *B* atoms and the *h*-sites show a depression.

Nevertheless, a numerical noise remains due to the approximation made, which induces the fuzzy aspect of the image. The amplitude of the numerical noise is about 0.01 Hz on the frequency shift.

In the repulsive regime, the tip is scanned with a height *H*_set_ = 2.75 Å, and the corresponding image is shown in Figure 2b. Notice that the input parameters are the same as those previously used, but now the frequency shift is positive. Because the slope of the curve of the interaction force is much more abrupt in the repulsive part than in the attractive part, the maximum of corrugation is larger and reaches 87 Hz. The numerical noise is hidden by such values of frequency shift and the image looks much sharper. During the oscillation and the scanning, the tip experiences a maximal force of about 1.43 nN.

The relative atomic contrast in the two images at constant height in the attractive and repulsive regimes remains the same (Figure 1b): The *A* atoms appear brighter than the *B* sites. Indeed, the images exhibit a honeycomb pattern, with the most attractive and the least repulsive force above the hollow site in Figure 2a and in Figure 2b, respectively. This is in qualitative agreement with experiments [38,40,42–43] and calculated results [38,44]. Quantitative comparison may be tricky because parameters are different (working parameter set, reactive or inert tip, etc.). In [38], a tip–sample interaction model is based on a Lennard-Jones potential and gives similar results if one compares the scan line in Figure 2d. Of course, such a pairwise potential (Lennard-Jones or Buckingham potential) is not able to describe a reactive tip, and the contrast is explained in terms of the Pauli repulsion in the repulsive region.

For the constant-frequency-shift mode at Δ*f*_set_ = −13 Hz, the result in the frozen-atoms mode is shown in Figure 2c. Here too, the tip explores the attractive range of the tip–surface interaction force with *H* around 4.58 Å and the tip experiences a minimal force of about −0.5 nN. One can see a contrast inversion compared to the previous cases in the constant-height mode (see Figure 1b and the corresponding table) but the bright spot above the hollow site has the same physical origin as previously, and it reflects the most attractive force as well. It is interesting to note that the maximum of corrugation is extremely small, about 0.004 Å which shows the consistent stability of the numerical distance controller. One may note that the chosen model of interactions gives a minimal Δ*f* of about −14 Hz. To keep the tip in the attractive regime and to avoid an instable regime in which the controller is not able to prevent a tip crash on the surface, we have taken the Δ*f*_set_ value mentioned above. Even if the corrugation is very low to be easily measured experimentally, one sees the difference between the *A* and *B* top sites and these results are qualitatively in agreement with experiments reported in the literature.

The results of the free-atoms mode at constant height (*H*_set_ = 2.75 Å) are shown in Figure 2d through a scanline above the graphite surface (see inset) corresponding to a condition at *T* = 4.9 K. Compared to the frozen-atoms result, the dynamic behavior induces a diminution of the corrugation (around 13 Hz) and a slight lateral shift due to the small motions of the carbon atoms during the scanning. Even a tiny out-of-plane displacement of the graphite atoms (<0.05 Å) generates a variation of about 90 Hz due to the abrupt slope of the Δ*f*(*H*) curve in the repulsive zone.

### Supported graphene on a silicon carbide substrate

**Figure 3:**
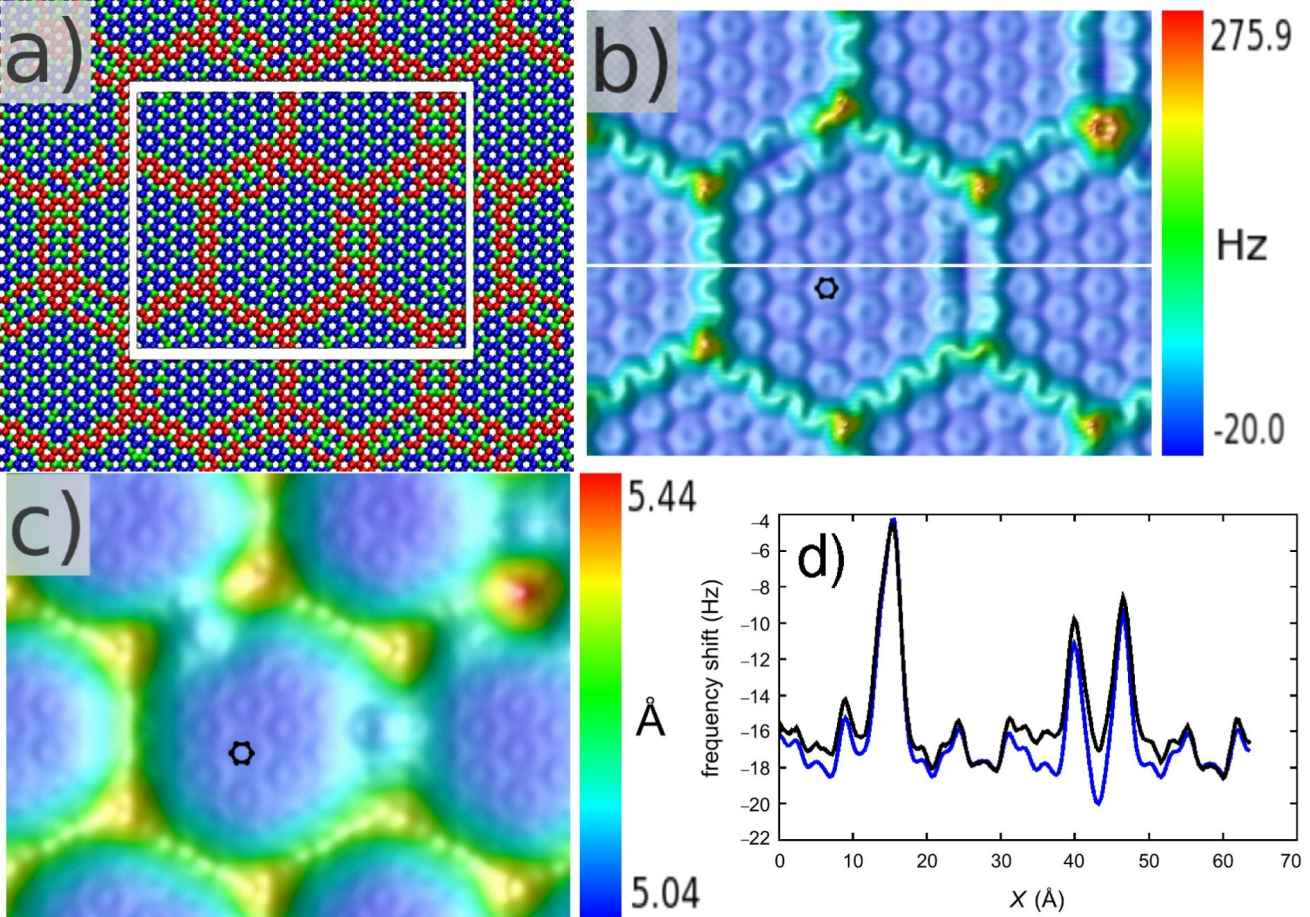
(a) Atomic structure of the buckled graphene on SiC with the height of graphene atoms classified in three categories: red, blue and green are the colors for the highest, the intermediate and the lowest carbon atoms, respectively. (b) Calculated graphene FM-AFM image with *H*_set_ = 3.8 Å along the white box in (a). The Δ*f* corrugation is 295.9 Hz. The image size is 63.9 × 50.1 Å^2^. The small black hexagon corresponds to a carbon ring of the surface. (c) FM-AFM calculated graphene image with Δ*f*_set_ = −12.5 Hz along the white box in (a). The height corrugation is 0.4 Å (5.44 Å to 5.04 Å). The image size is 63.9 × 50.1 Å^2^. (d) Scanlines above the white line in (b) for the frozen-atoms (blue) and free-atoms (black) regimes.

We consider here a graphene sheet on a Si-terminated 6H-SiC surface (5284 C atoms for the graphene sheet and three SiC layers for the substrate with 1332 Si and 1332 C atoms each giving in total a system of 13276 atoms). First, one has to consider the relaxation of the graphene layer with respect to the atomic structure of the substrate. By performing a full energy minimization of the system with DL_POLY-4 using periodic boundary conditions and with a Tersoff potential to connect the graphene and the SiC substrate, we found a buckling of the graphene sheet that is due to the incommensurability between the graphene and the SiC surfaces. The results shown in Figure 3a are similar to those obtained by DFT [74] or by using a more sophisticated empirical potential [110]: A quasi-hexagonal superstructure with a 6 × 6 periodicity is revealed with more or less long edges and a corrugation of about 1.2 Å. Such a soft corrugation of the moiré patterns is an interesting system for the n-AFM.

We performed FM-AFM calculations at constant height with *H*_set_ = 3.8 Å and at constant frequency shift with Δ*f*_set_ = −12.5 Hz after relaxation of the graphene layer on SiC. The results are presented in Figure 3b and Figure 3c, respectively. On both images in the frozen-atoms mode, one recognizes the graphene reconstruction with distinct edges, and sharp nodes at the crossing points of the edges. Indeed, the Δ*f* corrugation ranges from −20.0 Hz to 275.9 Hz in Figure 3b. This clearly indicates a rather strong repulsive regime at some points (yellow-red) due to the reduction of the tip–atom surface distance. Moreover, one can easily distinguish the long edges, which are two rows of C atoms higher than the narrow ones. The 6 × 6 periodicity was observed in noncontact mode AFM [111] but with smoother edges obtained in the constant-frequency-shift mode. In the attractive regime shown in Figure 3c, we observe a corrugation of 0.4 Å which may be measurable experimentally. Notice that there is no contrast inversion for the graphene hexagons on the ridges, whereas there is an inversion for hexagons in the center of the superstructure (Figure 1a and the corresponding table). This arises from a crossing between Δ*f*(*H*) curves as shown in Figure 1. At the center of the superstructure, one recovers the previous case of the graphite surface. The red (green) curve in Figure 1b could illustrate a tip–surface approach curve above a top (hollow) site: In the constant-frequency-shift mode, it is mandatory to work with a Δ*f*_set_ higher than the minimum Δ*f* (the red curve in the figure). As illustrated in Figure 1b, there is no intersection between the two typical curves. This thus implies a contrast inversion. On the contrary, and with the same requirement for the Δ*f* setpoint, if Δ*f*(*H*) curves have a crossing point, as illustrated in Figure 1a, there is no contrast inversion. In this figure, the red characteristics should represent an approach curve above a surface atom that sits slightly out of the plane compared to the green one, corresponding to an approach above an atom in the surface plane. This shift can occur due to the local relaxation of the carbon atom network, as is the case for the graphene ripples.

To go further, one needs to estimate the actual influence of the tip and of the temperature at *T* = 4.9 K in the free-atoms mode. By comparing the scan lines presented in Figure 3d, one sees that the system governed by MD exhibits, in some respects, a lower corrugation in the large ridge zones but a similar signal around the middle of the superhexagon. It appears also that the displacement of carbon atoms under these conditions is not homogeneous regarding their positions in the quasi-hexagonal superstructure. For rings on the higher ridge and at the center of the superhexagon, the mobility is reduced at *T* = 4.9 K. This is not the case for the other carbon rings, which induce a change in the frequency shift signal (almost 2 Hz at this temperature). This effect could be related to the local constraints of the carbon rings in the buckled graphene sheet.

### Graphene nanoribbon edges

In the recent literature in the graphene community, there is a vivid interest in graphene nanoribbons (GNR), because one may tune their electronic structure through chemical edge modification. Before reaching this stage, precise characterization of the structure of the edges has to be tackled experimentally by transmission electronic microscopy, scanning tunneling microscopy (STM) or calculations [79,82,112–118]. Generally, GNRs show a zigzag or armchair configuration. It was also demonstrated that the zigzag edge may reconstruct to a configuration with Stone–Wales-like defects consisting of alternate pairs of pentagons and heptagons. Nevertheless, a recent theoretical contribution shows that the zigzag edges are found to be dominant for graphene nanoribbons obtained with proper etching [119].

**Figure 4:**
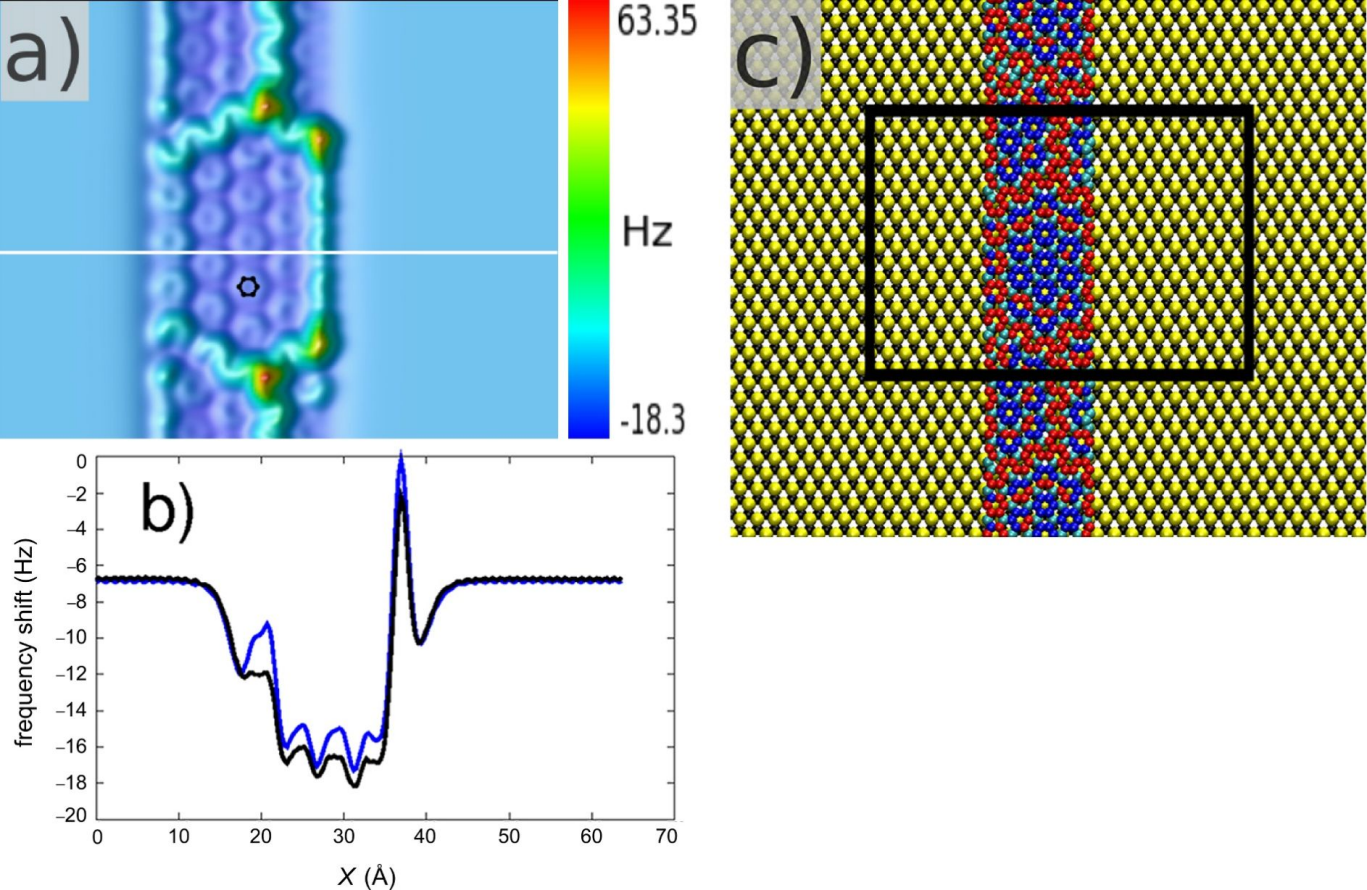
(a) FM-AFM calculated image of a graphene nanoribbon with *H*_set_ = 3.8 Å. The Δ*f* corrugation is 81.68 Hz. The image size is 63.9 × 50.1 Å^2^, which corresponds to the black area in (c). (b) Scan lines above the white line in (a) for the frozen-atoms (blue) and free-atoms (black) regimes. (c) Atomic model of the reconstructed GNR (same color code as in Figure 3d) on the SiC substrate (yellow, silicon atoms; black, carbon atoms).

Actually, as far as we know, there are no experimental FM-AFM imaging studies that reveal the edge structures of GNR. Nevertheless, some STM images succeed in identifying the edge conformation, although with a mixing of structural and electronic contributions [79]. As we are mainly interested in the capability of the n-AFM to image a GNR deposited on a SiC surface, we chose to start calculations with a GNR exhibiting a pristine zigzag edge [119]. The GNR consists of 684 carbon atoms forming a ribbon with a width of 18.38 Å. The SiC substrate is the same as previously used and the total system has 8676 atoms. Periodic boundary conditions are imposed along the main axis of the GNR. After a full energy relaxation, the GNR is slightly buckled and similar patterns to those seen in the graphene layer are obtained (Figure 4c). These patterns were also observed experimentally by STM on GNR or on graphene quantum dots [79,114–115]. A calculated FM-AFM image acquired with the n-AFM in the frozen-atoms regime is shown in Figure 4a. Here, the setpoint is *H*_set_ = 3.8 Å and the Δ*f* corrugation is 81.7 Hz.

At this setpoint, it is not possible to resolve the atomic structure of the SiC substrate, and the GNR appears globally darker due to the increase in attraction below the tip. If one compares the extremal values of the graphene and the GNR images, one remarks that the maximum Δ*f* is more than four times smaller for the GNR. It means that repulsion is less important due to smaller deformations of the network of carbon atoms in the GNR. This is consistent with the reduced size of the GNR in which the mechanical constraints are less important at the borders. One may also notice that the stability of the n-AFM is satisfactory during the scanning. Indeed, the tip oscillator experiences, first, a geometrical step due to the presence of the GNR on the SiC surface (the average distance between the GNR and the SiC surface plane is about 2.1 Å), and second, an interaction step between the bare SiC surface and the “SiC substrate plus the GNR”. These two steps are well accepted by the numerical DC of the n-AFM.

Finally, we compare the frozen-atoms and the free-atoms regimes along a scanline above the GNR in Figure 4b. The carbon atoms relax more freely generating a larger shift than in the case of graphene (1.5–2.0 Hz). One observes that the presence of the tip locally affects the structure at 4.9 K. It should also be mentioned that the lateral extension of the tip apex has a rather limited influence on the atomic behavior due to the limited size of the tip cluster. In order to take into account the interaction due to the lateral facets of the whole probe, one either discretizes the tip body by small volume elements and calculates a pairwise potential between each element and the atoms of the sample [120], or one adapts a self-consistent formalism to calculate the interactions between a dielectric probe of arbitrary shape and a corrugated surface [121–123].

## Conclusion

We have proposed calculated images with the help of a numerical AFM (n-AFM) working in the FM-AFM mode. This n-AFM is a reliable numerical tool to address different conditions of use, from large to small (qPlus) amplitudes, either at constant height or at constant frequency shift. Moreover, the coupling of a molecular dynamics module allows us to take into account an external temperature as well as the mechanical pressure of the tip during the sample scanning. We have shown three examples on graphitic structures: (i) a flat graphite surface, (ii) smooth corrugated ripples of a graphene sheet relaxed on a silicon carbide substrate, and (iii) a corrugated transition of a graphene nanoribbon supported by a SiC surface. Improvements remain to be made for the prospective study of single molecule imaging and/or manipulation processes and related physical problems, such as dissipation [66,124–125] and the influence of noise perturbations [126].
